# The National Lung Matrix Trial: translating the biology of stratification in advanced non-small-cell lung cancer

**DOI:** 10.1093/annonc/mdv394

**Published:** 2015-09-25

**Authors:** G. Middleton, L. R. Crack, S. Popat, C. Swanton, S. J. Hollingsworth, R. Buller, I. Walker, T. H. Carr, D. Wherton, L. J. Billingham

**Affiliations:** 1 Institute of Immunology and Immunotherapy, University of Birmingham, Birmingham; 2 Department of Oncology, University Hospitals Birmingham NHS Foundation Trust, Birmingham; 3 Cancer Research UK Clinical Trials Unit, University of Birmingham, Birmingham; 4 Department of Medicine, Royal Marsden NHS Foundation Trust, London; 5 The Francis Crick Institute, London; 6 UCL Cancer Institute, CRUK Lung Cancer Centre of Excellence, London; 7 Innovative Medicines Oncology, AstraZeneca, Cambridge, UK; 8 Pfizer Oncology, Pfizer, San Diego, USA; 9 Strategy and Research Funding, Cancer Research UK, London, UK

**Keywords:** National Lung Matrix Trial, non-small-cell lung cancer, stratified medicine, adaptive trial design, Umbrella Trial

## Abstract

**Background:**

The management of NSCLC has been transformed by stratified medicine. The National Lung Matrix Trial (NLMT) is a UK-wide study exploring the activity of rationally selected biomarker/targeted therapy combinations.

**Patients and methods:**

The Cancer Research UK (CRUK) Stratified Medicine Programme 2 is undertaking the large volume national molecular pre-screening which integrates with the NLMT. At study initiation, there are eight drugs being used to target 18 molecular cohorts. The aim is to determine whether there is sufficient signal of activity in any drug–biomarker combination to warrant further investigation. A Bayesian adaptive design that gives a more realistic approach to decision making and flexibility to make conclusions without fixing the sample size was chosen. The screening platform is an adaptable 28-gene Nextera next-generation sequencing platform designed by Illumina, covering the range of molecular abnormalities being targeted. The adaptive design allows new biomarker–drug combination cohorts to be incorporated by substantial amendment. The pre-clinical justification for each biomarker–drug combination has been rigorously assessed creating molecular exclusion rules and a trumping strategy in patients harbouring concomitant actionable genetic abnormalities. Discrete routes of pathway activation or inactivation determined by cancer genome aberrations are treated as separate cohorts. Key translational analyses include the deep genomic analysis of pre- and post-treatment biopsies, the establishment of patient-derived xenograft models and longitudinal ctDNA collection, in order to define predictive biomarkers, mechanisms of resistance and early markers of response and relapse.

**Conclusion:**

The SMP2 platform will provide large scale genetic screening to inform entry into the NLMT, a trial explicitly aimed at discovering novel actionable cohorts in NSCLC.

**Clinical Trial ISRCTN:**

38344105.

## Abstract (precis)

The National Lung Matrix Trial is currently the largest stratified medicine study in lung cancer. Utilizing a next-generation sequencing screening platform and an adaptive umbrella trial design, we will explore the activity of multiple biomarker/targeted therapy options in order to expand the precision medicine opportunities for patients with non-small-cell lung cancer.

## introduction

The management of patients with non-small-cell lung cancer (NSCLC) has been transformed in the past 10 years. The identification of EGFR-activating mutations as a predictive biomarker for the use of EGFR tyrosine kinase inhibitors ushered in the era of stratified medicine in NSCLC [1]. Only 4 years elapsed between the description of *EML4-ALK* fusions [2] and the registration of crizotinib for treatment of *ALK* fusion-positive disease. Alongside, these therapeutic advances have been a change in the regulatory landscape; the provisional registration of crizotinib was based on high signals of activity in non-randomized, single-arm studies [3]. A series of publications culminating in the data from The Cancer Genome Atlas (TCGA) for both adenocarcinoma and squamous cell lung cancer have considerably widened the number of potentially treatable targets, albeit in small molecularly defined patient cohorts [4, 5]. Efficient testing of drug–biomarker combinations is necessary in order to unlock the true potential for stratified medicine for NSCLC. The National Lung Matrix Trial (NLMT), funded by Cancer Research UK in partnership with AstraZeneca/MedImmune and Pfizer, includes many of the potentially actionable molecular aberrations identified in NSCLC. We describe the overarching design of the study and the selection of agents according to molecular abnormality.

## methods

The NLMT is a multi-arm non-randomized non-comparative phase II umbrella trial in which patients are allocated to the appropriate targeted therapy according to the molecular genotype of their cancer. The trial includes a common set of outcome measures for all molecularly defined cohorts with flexibility to select a cohort-specific primary end point. In most cases, response rate is the primary outcome but for agents whose mode of action is likely to be principally cytostatic, progression-free survival (PFS) is preferred. Although randomized trials make it possible to tease out the predictive and prognostic effects of putative biomarkers for therapies, we are looking here for robust signals of activity such as one would expect from a bona fide targeted therapy. For example, the recent demonstration of a 72% response rate and a 19-month median PFS in patients treated with crizotinib harbouring *ROS* fusions [6] is very clear evidence that this drug works in this cohort of NSCLC patients. Such data, in a very small segment of NSCLC, begin to challenge both the practicality and the need for the traditional randomized trial approach to obtain drug approvals. Indeed, with very small target populations, it will become essential for regulatory science to rapidly evolve if we are to realize the magnitude of the opportunity for precision medicines in cancer.

There is an option within the trial protocol to test any of the given targeted therapies on biomarker-negative patients (i.e. those with no actionable genetic change) if there is evidence of significant activity in the biomarker-positive population. This allows validation of the specificity of the putative biomarker for that drug but may also detect biomarker-negative patients who have impressive responses to the drug and whose tumours can then be analysed to detect abnormalities that may be additional important positive predictive biomarkers of that drug.

The NLMT will be run at all 18 UK Experimental Cancer Medicine Centres (ECMC) with each centre operating a hub and spoke model with patients being referred in from nearby hospitals to the centre. The pace of advances in stratified medicine in lung cancer is such that signal of activity programmes must be nimble, flexible and able to respond promptly to new biomarkers drug combinations being considered. As such, the trial allows for new arms to be entered via substantial amendment, if the international expert review panel and Trial Management Group are convinced of the strength of the pre-clinical data supporting the clinical combination of the biomarker and targeted agent. This will significantly reduce the time from concept to clinical study.

One of the limitations for the development of targeted agents in small patient populations has been the conventional approach to tumour testing which could be described as ‘one drug–one test.’ This limitation will be overcome in the NLMT by implementing an umbrella trial design. This approach will facilitate a transition from a drug-centred approach that has asked ‘can a specific therapeutic agent be given to a patient’, to a patient-centred molecular testing approach which allows the treating physician to ask ‘what is best therapeutic agent for my patient?’

Screening of patients' tumour biopsies through the Stratified Medicine Programme 2 is performed on a next-generation sequencing (NGS) panel developed and validated by Illumina, carried out in one of three dedicated genotyping centres (Technology Hubs). At present, 28 genes are interrogated but the platform is adaptable to allow new genomic biomarkers to be added. Alignment of tumour DNA reads against germline is mandatory. Careful examination of genetic databases, pre-clinical and clinical data have generated a comprehensive tiering system which ensures that only oncogenically pertinent abnormalities are actioned. Table 1 represents the matrix of molecular cohorts with their targeted agents, together with the predicted frequency of these abnormalities.

**Table 1.** NLMT molecular cohorts and estimated prevalence rates

Molecular cohorts and initial estimated prevalence rates		AZD4547	AZD2014	Palbociclib	Crizotinib	Selumetinib + docetaxel	AZD5363	AZD9291
		Arm A	Arm B	Arm C	Arm D	Arm E	Arm F	Arm G
A1: FGFR2/3 mutation—NSCLC [4, 5]	ADC <1.0% SCC 4.0%	✓						
B1: TSC1/2 mutation—NSCLC [4]	ADC <1.0% SCC 2.7%		✓					
B2: LKB1 mutation—NSCLC [4, 5]	ADC 8.8% SCC 1.6%		✓					
C1: Proficient Rb and p16 loss—SCC [4]	SCC 29.0%			✓				
C2: Proficient Rb and p16 loss—ADC [4]	ADC 19.6%			✓				
C3: Proficient Rb and CDK4 amplification—NSCLC [4, 5]	ADC 7.0% SCC <1.0%			✓				
C4: Proficient Rb and CCND1 amplification—NSCLC [4, 5]	ADC 5.0% SCC 12.0%			✓				
C5: Proficient Rb and KRAS mutation—ADC [5]	ADC 25.8%			✓				
D1: Met amplified—NSCLC	ADC 2.7% SCC 1.4%				✓			
D2: ROS1 rearranged—NSCLC [6]	ADC 1.7% SCC <1.0%				✓			
E1: NF1 mutation—SCC [4]	SCC 5.8%					✓		
E2: NF1 mutation—ADC [4]	ADC 4.6%					✓		
E3: NRAS mutation—ADC [7]	ADC 0.7%					✓		
F1: PIK3CA mutation—SCC [8]	SCC 11.0%						✓	
F2: PIK3CA amplification—SCC [4]	SCC 15.0%						✓	
F3: PI3K/AKT deregulation								
PI3KCA mutation and amplification—ADC [8]	ADC 2.0%						✓	
PTEN mutation and loss (ADC) [5]	ADC 3.0%							
AKT1 mutation (NSCLC) [4, 5]	ADC 0.5% SCC 0.5%							
F4: PTEN loss and mutation—SCC [4]	SCC 20.0%						✓	
G1: EGFR mutation and T790M + NSCLC	ADC 8% SCC <1%							✓

## the rationale supporting the biomarker/drug combinations in the National Lung Matrix Trial

### AZD5353

AZD5363 is potent ATP-competitive AKT inhibitor with IC_50_ <10 nM for all three AKT isoforms. Using a cut-off of GI_50_ <3 µm, 23% of a large cell-line panel were sensitive to inhibition by AZD5363 and three quarters of these had *PIK3CA* mutations, *PTEN* loss or *HER2* amplification [9]. *In vivo* activity was demonstrated in both *PIK3CA* mutant and *PTEN* inactivated xenograft models. In a study using the PI3K inhibitor GDC-0941, 3/3 *PIK3CA* amplified cell lines (trial arm F2) were highly sensitive to inhibition and there was no co-occurrence of other obvious oncogenic drivers [10].

Each separate mechanism resulting in AKT activation is treated as a separate cohort. This is an important design feature of the trial. It is unlikely that the activities of targeted agents will be the same for each mechanism of deregulation and treating them all in one cohort may miss the granularity of this response differential. Furthermore, mechanisms of resistance may vary in patients who have an initial response to therapy according to the mechanism of initial AKT activation. Discrete cohort testing allows the specifics of resistance in each molecular cohort to be defined.

**Figure 1.**
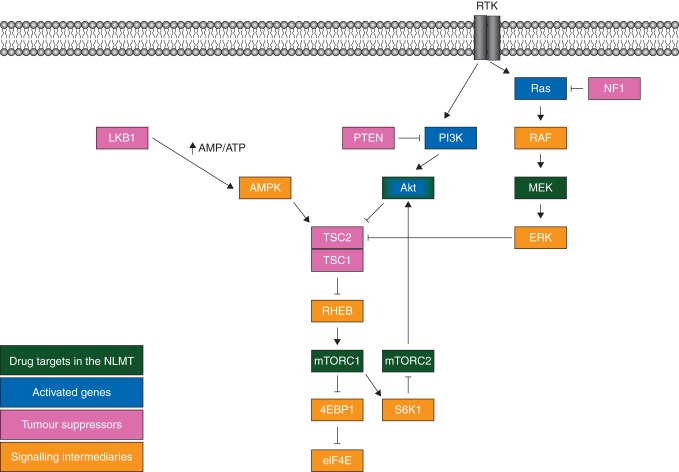
Key signalling pathways targeted in the National Lung Matrix Trial.

All patients' tumours in this treatment arm will need to be proven to be *KRAS* wild type. KRAS was shown to be a negative predictive biomarker for AZD5363 response [9]. There is collaborative inactivation of the translational repressor 4E-BP1 by both AKT and ERK signalling [11], ERK directly inactivates TSC2 by phosphorylation [12]. Thus, cells with dual AKT activation and *RAS* mutation may still inhibit 4E-BP1 even if AKT is inhibited (Figure 1).

### AZD4547

AZD4547 is potent inhibitor of FGFR 1, 2 and 3 with IC_50_ values of 0.2, 2.5 and 1.8 nM, respectively [13]. Initial data in NSCLC patients with somatic *FGFR* amplifications treated with AZD4547 showed modest efficacy (8% PR, 29% SD) but failed to meet the primary end point for continuation [14]. Not all detected *FGFR* mutations will be eligible. Liao et al. engineered NIH3T3 cells to express the range of separate mutations represented in the squamous cell lung cancer TGCA [15]. Only mutations in the extracellular binding domain, which mediated ligand independent receptor dimerization and activation, and mutations in the intracellular tyrosine kinase domain were transforming and tumorigenic. Those in the trans-membrane spanning region and the terminal portion of the molecule were not transforming, and these two latter will be excluded from testing with the drug.

### AZD2014

AZD2014 is an ATP-competitive, selective mTOR kinase inhibitor targeting both mTORC1 (rapamycin-sensitive) and mTORC2 (rapamycin-insensitive) complexes [16, 17]. AZD2014 is molecularly different from rapalogues and achieves more profound mTORC1 inhibition, in particular, inhibiting phosphorylation of the rapamycin-insensitive site on 4E-binding protein 1 (4E-BP1) (T37/46). AZD2014 also inhibits mTORC2 and has a broader range of growth inhibitory activity *in vitro* across tumour types compared with rapalogues. mTORC1 inhibition reduces S6K1 activation which negatively represses mTORC2; thus, mTORC1 inhibition releases mTORC2 from S6K1-mediated inhibition and activates AKT via mTORC2-mediated phosphorylation of Ser473 [18] (Figure 1). Inhibition of mTORC2 is therefore important because it prevents the activation of AKT via phosphorylation on Ser473 consequent upon inhibition of mTORC1 by rapalogues. Patients with *TSC1*, *TSC2* or *LKB1* (*STK11*) tumour mutations will be eligible for treatment with AZD2014.

The TSC1/2 heterodimer is a GTPase-activating protein which maintains Rheb in its inactive GDP-bound form. Rheb is the upstream activator of mTOR. Hence, when TSC1 or TSC2 activity is lost through mutation, Rheb becomes activated which in turn activates mTOR. TSC mutant cell lines were highly sensitive to inhibition by AZD2014 with all mutants having a GI50 of <1 µM and 8/10 TSC1 mutants having a GI50 <200 nM (AstraZeneca, internal data). A recent clinical study has demonstrated that mTOR inhibitors have significant activity in TSC mutant disease. A patient with anaplastic thyroid cancer who relapsed after surgery and subsequent chemoradiation had an 18-month response to the mTORC1 inhibitor everolimus. Sequencing this patient's tumour DNA revealed a *TSC2* mutation [19].

Loss of LKB1 function phenocopies TSC2 mutation (Figure 1). LKB1 is critical in the activation of AMPK in situations of cellular energy stress, such as hypoglycaemia, which increases the AMP/ATP ratio [20]. AMPK activates TSC2, so when LKB1 is lost, TSC2 is less efficiently activated. mTOR activation causes activation of HIF-1α and this upregulates lysyl oxidase (LOX) activity. This is a key enzyme stabilizing the extracellular matrix via oxidation of lysine residues on collagen and elastin, and such stabilization is important in mediating the process of hypoxia-induced metastasis. Knock-down of LOX in *LKB1* mutant cells resulted in reduced anchorage independent cell growth and migration [21]. The LOX inhibitor BAPN had no activity in *LKB1* wild-type models but reduced tumour number and volume in LKB1 mutant models.

### palbociclib

Palbociclib is a highly selective inhibitor of CDK4/6 kinase activity (IC_50_ = 11 nM; Ki = 2 nM. Palbociclib has selectivity for CDK4/6, with little or no activity against a large panel of 34 other protein kinases [22].

**Figure 2.**
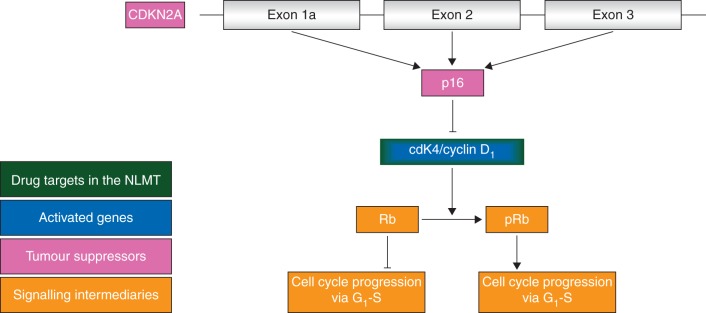
The CDKN2A–CDK4 axis.

p16, a product of the *CDKN2A* locus, is the key inhibitor of the cyclinD1/CDK4 heterodimer (Figure 2). When this dimer is activated, either by homozygous deletion of *CDKN2A* or amplification of *CDK4* or *Cyclin D1*, Rb becomes phosphorylated. When further phosphorylated by CDK2/Cyclin E, this removes the inhibitory activity of RB upon E2F1, resulting in the liberation of E2F and the passage of cancer cells through the G1/S checkpoint. In patients whose cancers harbour one of these three molecular abnormalities and would be eligible for treatment with palbociclib, it must be demonstrated that their cancer harbours no concomitant loss of Rb function. In a panel of ovarian cancer cell lines, Rb-proficient cells with low p16 expression (by message or protein expression) were the most sensitive to palbociclib, and no other analysed biomarkers pertaining to CDK4/6 signalling were informative [23]. Expression profiles of cells classified by response to palbociclib demonstrated 117 differentially expressed genes between sensitive and resistant lines: *CDKN2A* was the most significant gene.

*KRAS* mutant adenocarcinoma represents a significant molecular cohort of lung adenocarcinoma. It was demonstrated that loss of *CDK4* activity was synthetically lethal with *KRAS* mutation in lung adenocarcinoma [24]. This effect was specific: it was not seen *in vivo* with knock-down of the other CDKs and was not seen in other mutant KRAS expressing tissues including the pancreas. Palbociclib was shown to have significant activity in *KRAS* mutant genetically engineered mouse models. The mechanism of synthetic lethality appeared to be due to the re-induction of senescence. *KRAS* mutation causes oncogene-induced senescence and for the development of the full malignant phenotype, *KRAS* mutant cells must bypass senescence. Thus, CDK4 inhibition is expected to abrogate this bypass in patients whose lung cancers harbour an intact Rb signalling pathway and a *KRAS* mutation. Activated *RAS* induces the formation of senescence-associated heterochromatin foci by activating GSK3β which phosphorylates the histone chaperone HIRA facilitating its localization to PML nuclear bodies [25]. *AKT* activation inhibits RAS-mediated oncogene-induced senescence in part through the inhibitory phosphorylation of GSK3β at serine 9 [26]. Thus, a molecular exclusion for this arm is concomitant abnormalities which result in AKT activation, such as PIK3CA mutation, PTEN loss or AKT mutation.

### crizotinib

Crizotinib is a potent inhibitor of both MET and ROS1. There is already clear evidence of activity of crizotinib in patients with tumour *MET* amplification [27] or *ROS1-*rearranged lung cancer [6]. These arms as well as adding to the global database of preliminary activity of this drug in these cohorts will also provide access for patients with these actionable molecular aberrations.

### AZD9291

About 50%–60% of patients with *EGFR* mutation-positive tumours progressing after first-line treatment on an EGFR TKI become resistant via the acquisition of the secondary gatekeeper mutation T790M. AZD9291 is an oral, potent, selective, irreversible inhibitor of both EGFR-TKI-sensitizing and T790M resistance mutations. In 127 assessable patients with T790M disease, a response rate of 61% and median PFS of 9.6 months was observed [28]. Patients who have received a first-line EGFR TKI will be invited to have a repeat biopsy on progression and, if their cancers have acquired the T790M mutation, treatment with AZD9291 will be offered.

### selumetinib and docetaxel

Selumetinib is a potent, selective, allosteric MEK inhibitor. In a randomized phase II study, it was demonstrated that a combination of selumetinib plus docetaxel significantly improved response rate and PFS when compared with docetaxel alone as second-line therapy for patients with *KRAS* mutant adenocarcinoma of the lung [29]. This combination is currently in phase III in this setting. In the lung adenocarcinoma TCGA, *NF1* mutations were found to be significantly represented in cancers not harbouring abnormalities of *RAS*, *RAF* or receptor tyrosine kinases [11]. The authors specifically identified *NF1* as an important driver event in oncogene-negative adenocarcinoma. *NF1* is a RAS GAP (Figure 1). It restricts *RAS* activation by catalysing the intrinsic GTPase activity of *RAS*. Thus, when *NF1* is inactivated by mutation, *RAS* becomes locked in its GTP-bound form resulting in constitutive activation. Hence, *NF1* loss phenocopies *KRAS* mutation and patients with such cancers can also be considered a suitable molecular cohort to treat with the selumetinib/docetaxel combination, which has proven benefits in *KRAS* mutant disease. There is good clinical evidence for the importance of MEK signalling in *NF1* mutation. Patients with germline inactivation of NF1 develop plexiform neurofibromas (PNs); in a cohort of PN patients aged 3–18, all patients treated with single-agent selumetinib demonstrated volumetric reduction in the size of their PNs [7].

In patients whose tumours harbour NRAS mutations, there appear to be no other obvious driver mutations [30]. 5/6 *NRAS* mutant lung cancer cell lines were sensitive to single-agent selumetinib and MEK signalling appeared to be significantly more important than *PI3K/AKT* activation given the lack of effect of GDC-0941 in these *NRAS* mutant lung cancer cell lines. Thus, we will treat patients with *NRAS* mutant tumours as a separate cohort with this combination.

### no actionable mutation arm

A secondary objective of the trial is to offer a therapeutic option for patients with successful screening in the trial but without specific eligibility for one of the targeted genomic aberrations at that moment in time. The first in a planned series of drugs that we are testing in this cohort is MEDI4736, an anti-PDL1 monoclonal antibody with clear evidence of activity in patients with NSCLC [31].

## concluding remarks

The NLMT is an ambitious adaptive programme which seeks to increase the number of actionable genetic abnormalities in NSCLC. Key translational components include voluntary pre-treatment biopsies to identify potential predictive biomarkers post-treatment biopsies to ascertain mechanisms of resistance, and the development of PDX models wherever possible. ctDNA is being collected pre-, during and post-treatment. Repeat biopsies can be difficult to obtain: the recent identification of the C797S mutation in the plasma of patients with T790M mutation-positive disease treated with AZD9291 in second line or beyond demonstrates the utility of liquid biopsies to identify mechanisms of resistance to targeted therapies [32].

Ensuring the sustainability of NLMT is important in order to maximize the information that can be obtained from this programme. We are in discussion with a number of other potential pharma partners and have already discussed with our current pharma collaborators plans for treatment of patients with p53 loss and patients with rarer but potentially actionable mutations such as those with ATM loss and exon 14 slipping mutations in *MET*. Finally, the NLMT is clearly not the only such programme in this therapy space. The NCI-MATCH co-operative group trial currently has 10 arms: some cohorts are in common with NLMT but NCI-MATCH also covers BRAF mutations (dabrafenib/trametinib), HER2 mutations (afatinib), HER-2 amplifications (Ado-trastuzumab emtansine) and c-kit mutations (sunitinib). Integrating the data from this trial together with data from NLMT, other key trials such as SAFIR-02 (NCT02117167) and LUNGMAP (NCT02154490), and further data arising from national genomic screening initiatives such as The Lung Cancer Genome Project (Network Genomic Medicine, Cologne) and LC-SCRUM (Japan), will allow the development of a global database of outcomes on personalized medicines in NSCLC.

## funding

This work is supported by Cancer Research UK (C22436/A18392 and C11497/A19363), AstraZeneca/MedImmune and Pfizer (provision of Investigational Medicinal Products). Note that the Pharma Partners are providing core funding into the Stratified Medicine Programme in addition to the compounds.

## disclosure

GM receives research funding from AstraZeneca and Cancer Research UK. SP is non-compensated consultant to AstraZeneca and Pfizer. LB has received personal fees from Eli Lilly and Roche outside of the submitted work. All remaining authors have declared no conflicts of interest.
